# Physicochemical properties of Alisma starch

**DOI:** 10.3389/fnut.2025.1513814

**Published:** 2025-01-22

**Authors:** Fenxia Han, Yongqiang Wang, Hao Zhang, Sheng Zhang

**Affiliations:** ^1^School of Animal Science and Technology, Henan Institute of Science and Technology, Xinxiang, China; ^2^Shaanxi Research Institute of Agricultural Products Processing Technology, Xi'an, Shaanxi, China; ^3^College of Food Science and Engineering, Inner Mongolia Agricultural University, Hohhot, China

**Keywords:** Alisma, starch, physicochemical properties, crystal structure, pasting properties, gel strength

## Abstract

**Introduction:**

Alisma starch (AS) from Alismatis Rhizoma has potential applications but has been less studied compared to common starches like corn starch (CS) and potato starch (PS).

**Methods:**

We used scanning electron microscopy, X-ray diffraction, and rapid visco analysis to study the granule morphology, crystal structure, pasting properties, freeze -thaw stability, solubility, swelling degree, and gel strength of AS, CS, and PS.

**Results:**

AS has a lower starch content but higher amylose content than CS and PS. It has a smaller particle size and is A-type starch. Its pasting temperature and trough viscosity are higher, and its freeze -thaw stability is intermediate. Gel strength increases with concentration and shows no significant difference between 10% AS and 12% PS.

**Discussion:**

AS has good heat resistance, shear resistance, and gel strength, indicating potential for high-temperature processed foods. Future research should focus on its heat resistance mechanism and broader applications.

## 1 Introduction

Alismatis Rhizoma, the tuber of *Alisma orientale* (Sam.) Juz. in the family of Alismataceae, is distributed in temperate and subtropical regions all over the world, mainly in China, Japan, North America and Europe (1). Recognized for its substantial nutritional and medicinal value, it serves as a dual-purpose medicinal and food crop. Contemporary pharmacological investigations have unveiled a spectrum of pharmacological activities associated with Alismatis Rhizoma, encompassing diuretic, nephroprotective, hepatoprotective, hypolipidemic, anti-inflammatory, antitumor, hypoglycemic and antioxidant (2, 3). Clinically, it is often used for the treatments such as edema, oliguria, hyperlipidemia and other diseases (4). The chemical constituents of Alismatis Rhizoma mainly include triterpenes, diterpenes, sesquiterpenes, amino acids, polysaccharides, proteins, fatty acids, starch, among other constituents, with Alisma starch (AS) representing approximately 25% of the total components (5). Notably, current research in the realm of starch-related investigations has predominantly centered on corn starch (CS), potato starch (PS), and similar sources (6), with a dearth of studies elucidating the physicochemical properties of AS.

Starch, a carbohydrate extensively stored in plants, finds diverse applications in the realms of food, medicine, and cosmetics. Within the food industry, starch serves as a vital component utilized as a thickener, stabilizer, and emulsifier to enhance the sensory attributes and palatability of food products (7). In pharmaceutical field, starch is employed as auxiliary material in the production of pharmaceuticals, notably as fillers for capsules and coating agents for granules (8). Moreover, in the cosmetic industry, starch is harnessed for its properties in enhancing skin texture, providing skin hydration, and absorbing excess oil (9). To date, a comprehensive investigation encompassing AS, CS and PS remains unexplored. Consequently, the objective of the present study is to systematically examine the physicochemical properties of AS, including preparation methods, composition, granule morphology, crystal structure, pasting attributes, freeze–thaw stability, solubility, swelling capacity, and gel strength. A comprehensive comparative analysis of the three starches AS, CS and PS is poised to advance the extensive utilization of AS, foster pioneering advancements in related industries, and furnish a theoretical framework for the development and utilization of AS resources.

## 2 Materials and methods

### 2.1 Materials and chemicals

The Alismatis Rhizoma tubers were purchased from Shanghai Aladdin Biochemical Technology Co., Ltd. (Shanghai, China), while CS and PS were purchased from Hebei Zhaofa Food Co., Ltd. (Langfang, China). All other chemicals utilized in the study were of analytical grade.

### 2.2 Preparation of Alisma powder

Fresh and unblemished roots of Alismatis Rhizoma were carefully chosen, meticulously cleaned, and subsequently pulverized employing a pulverizer. To prevent denaturation of the Alisma powder due to overheating during the pulverization process, an intermittent method was employed: the pulverizer was operated for 1 min, followed by a 1-min pause, the cooling was assisted by cold air and repeated. Subsequently, the pulverized Alisma material was ground to pass through a 100-mesh sieve for subsequent utilization.

### 2.3 Preparation of AS

Referring to the method of Shrivastava et al. (10) with minor modifications, the prepared Alisma powder underwent a defatting process. Initially, the Alisma powder was immersed in n-hexane at a ratio of 1:10 (w/v) and subjected to agitation using a magnetic stirrer for 3 min at intervals of 1 h to facilitate fat extraction. The n-hexane solution was refreshed every 6 h, and this sequence of agitation was repeated thrice. Subsequently, the defatted Alisma powder was treated with 70% ethanol at a ratio of 1:5 (w/v) for 30 min, followed by centrifugation at 4,000 rpm/min for 15 min. The resulting supernatant was discarded, and this process was iterated until the supernatant appeared colorless, aiming to remove phenolic and pigmented substances. The decolorized Alisma powder was immersed in water at a ratio of 1:15 (w/v) with a pH maintained at 10. Subsequent to stirring with a magnetic stirrer at 500 rpm for 6 h, the resultant mixture was centrifuged at 4,000 rpm for 10 min, and the supernatant was discarded. The AS precipitate was then isolated, and its pH was adjusted to 7.0 with 1 mol/L HCl. The starch slurry was washed repeatedly with distilled water, lyophilized, and grinded through a 100-mesh sieve to obtain the final AS product.

### 2.4 Determination of starch and amylose content

The determination of starch content followed the method of Song et al. (11). A sample weighing 0.75 g was mixed with 50 mL of 1% hydrochloric acid, and the mixture was subjected to boiling in a water bath for 15 min before being rapidly cooled. Subsequently, 1 mL of 30% zinc sulfate solution was added and thoroughly mixed, followed by the addition of 1 mL of potassium ferricyanide solution and mix well, pour into a 100 mL volumetric flask and filter. The optical rotation value of the filtrate was determined using a WZZ-2S automatic polarimeter (INESA Physico-Optical Instrument Co., Ltd., Shanghai, China).

The determination of amylose content was conducted using a colorimetric method. Standard solutions of amylose and amylopectin at varying ratios of 1 mg/mL were prepared by mixing 2.5 mL of each solution with 20 mL of distilled water, adjusting the pH to 3.0, and adding 0.5 mL of iodine reagent. After thorough mixing, the volume was adjusted, and the solutions were allowed to stand away from light for 10 min. Subsequently, the absorbance at 620 nm was measured using a PERSEE TU-1810PC UV spectrophotometer (Beijing, China), and a standard curve was constructed based on these measurements. A precise amount of 50 mg of starch was weighed and mixed with 10 mL of 0.5 mol/L sodium hydroxide solution, followed by agitation in a boiling water bath for 15 min. The volume was then adjusted to 50 mL with distilled water. Subsequently, 2.5 mL of the resulting sample solution was withdrawn and combined with 20 mL of distilled water. The pH was set to 3.0, and 0.5 mL of iodine reagent was added before adjusting the volume. The solution was shielded from light for 10 min, and the absorbance was measured at a wavelength of 620 nm. The percentage of amylose was calculated according to the standard curve (12).

### 2.5 Characterization of starch particle morphology

The samples were evenly distributed on the sample stage with double-sided adhesive tape, followed by gold sputter coating. Subsequently, the morphology of the samples was examined at a magnification of 2000× using an FEI Quanta 200 Environmental Scanning Electron Microscope (Hillsboro, OH, United States) (13). The birefringence of starch granules was observed under polarized light by dropping 0.1% of the sample solution onto a slide and placing it on a BH200P polarizing microscope (Shanghai Sunny Hengping Scientific Instrument Co., Ltd., China) (14). Furthermore, the particle size distribution of the samples was determined using a BETTER BT-9300H laser particle size analyzer (Dandong, China) (15).

### 2.6 Determination of X-ray diffraction

A Bruker D8 Advance X-ray diffraction analyzer (Karlsruhe, Germany) was used for the recordings, and the measurement conditions were step-scanning method with a 2θ scanning range of 5° ~ 60°, a step size of 0.02^o^, and a scanning speed of 8^o^/min. The relative crystallinity of the samples was calculated using Jade6 software (Livermore, CA, 135 USA) (16).

### 2.7 Determination of pasting properties

Starch pasting Properties were determined using Rapid Visco Analysis (RVA). A 3.0 g starch sample was mixed with 25 mL of distilled water and analyzed in an RVA 4500 rapid viscometer (Stockholm, Sweden). The sample was kept at 50°C for 1 min, warmed to 95°C within 3.5 min, held for 3 min, then cooled to 50°C within 3.5 min, and held for 2 min. The changes in viscosity of the sample throughout this process were monitored and recorded (17).

### 2.8 Determination of clarity

The determination of clarity was conducted following the procedure outlined by Fan et al. A certain amount of starch sample was weighed to prepare a 1% (w/v) suspension, which was then heated and stirred in a boiling water bath for 15 min. Subsequently, the suspension was cooled to room temperature, and the transmittance was measured at 620 nm using a UV spectrophotometer, with distilled water serving as a reference. The transmittance value was utilized as an indicator of the clarity of the starch paste (18).

### 2.9 Determination of sedimentation volume

A specific quantity of starch sample was weighed and used to prepare a 1% (w/v) suspension. The suspension was placed in a boiling water bath and stirred for 15 min until completely dissolved. Subsequently, 50 mL of the starch paste was transferred to a measuring cylinder and allowed to stand for 24 h. The volume of sediment (mL) was then recorded (19).

### 2.10 Determination of freeze–thaw stability

A certain amount of starch sample was weighed to prepare a 6% (w/v) suspension, which was then subjected to heating and stirring in a boiling water bath for 15 min before cooling to room temperature (20). An appropriate amount of starch paste (W_1_) was frozen in a refrigerator at −18°C for 24 h, then removed and thawed, and subsequently centrifuged at 4,000 rpm for 15 min. Finally, the weight of the separated supernatant (W_2_) was recorded and the syneresis rate (SR) was calculated according to Equation (1):


(1) $$SR\;\left(\%\right)=\frac{W_{2}}{W_{1}}\times 100\%$$

Where W_1_ and W_2_ are the weights of separated supernatant and starch paste, respectively.

### 2.11 Determination of syneresis

A certain amount of starch sample was weighed to prepare a 4% (w/v) suspension, which was heated and stirred in a boiling water bath for 30 min for aging. Subsequently, an adequate volume of starch paste (W_1_) was refrigerated at 4°C for 24 h, then removed, centrifuged at 4,000 rpm for 15 min, and the weight of the supernatant (W_2_) was recorded. The syneresis rate (SR) was calculated based on these measurements, reflecting the extent of starch syneresis (21). Calculation of syneresis rate is the same as 2.10.

### 2.12 Determination of solubility and swelling degree

A specific quantity (M) of starch samples was weighed to prepare 5% (w/v) suspensions. These suspensions were individually subjected to heating at 55, 65, 75, 85, and 95°C in a water bath for 30 minutes with constant oscillation (22). After cooling to room temperature following the pasting process, the samples were centrifuged at 4,000 rpm for 15 min. The supernatant was then transferred into pre-weighed glass weighing dishes, dried in a 105°C oven to a constant weight to determine the mass of dissolved starch (M_1_). The remaining mass after centrifugation represented the mass of swollen starch (M_2_). The solubility (SOL) and swelling degree (SD) of starch were calculated according to the following Equations (2) and (3):


(2) $$SOL\;\left(\%\right)=\frac{M_{1}}{M}\times 100\%$$

(3) $$SD\;\left(\%\right)=\frac{M_{2}}{M\left(1-\mathrm{SOL}\right)}\times 100\%$$

M, M_1_, and M_2_ in the formula are the mass of starch, the mass of dissolved starch, and the mass of precipitate after centrifugation, respectively.

### 2.13 Determination of gel strength

Preparation of samples for the gel strength test involved weighing a specific amount of starch samples to prepare 8, 10, and 12% (w/v) suspensions. These suspensions were heated and stirred in a boiling water bath until the starch reached a viscous state, at which point the stirring was immediately ceased, and the suspension was sealed with plastic wrap. The starch was then heated continuously for 30 min to ensure complete pasting, followed by removal and cooling to room temperature. Subsequently, the samples were placed in a refrigerator at 4°C for 16 h to allow for the formation of stable starch gel sample (23).

The gel strength determination was conducted using a TA-XT Plus texture analyzer (Stable Micro Systems, Surrey, UK) equipped with a dedicated probe (P/0.5). The analysis was carried out in GMIA Gelation mode at a testing temperature of 25°C. The parameters were configured as follows: trigger force of 2 g, pre-test speed of 1.5 mm/s, mid-test speed of 1.0 mm/s, post-test speed of 1.0 mm/s, compression depth of 10 mm, auto-trigger enabled, and a distance of 4 mm (24).

### 2.14 Statistical analysis

Results are shown as mean ± standard deviation (n = 3). Significance analysis was performed using the Tukey method with a 95% confidence interval. Statistical analysis and graphical representation were conducted using Origin 2018 software.

## 3 Results and discussion

### 3.1 Composition of AS

Significant differences (*p* < 0.05) in starch content and amylose content were observed in AS, CS, and PS, as shown in Table 1. The starch content of AS (82.37 ± 0.59%) is significantly lower than that of CS (88.62 ± 1.51%) and PS (86.72 ± 0.69%). The amylose content of AS (27.20 ± 0.22%) is significantly higher than that of PS (24.47 ± 0.36%), aligning with the findings of Tian et al. (25). This phenomenon may be attributed to the activity of granule-bound starch synthase in plants, which catalyzes the synthesis of amylose (26). Starch consists of amylose and amylopectin, the ratio of which is closely related to the starch source and also governs the starch’s characteristics, thereby influencing its capacity to form complexes with other compounds. Amylose supplementation reduces the digestibility of starch in foods and serves as a rich source of resistant starch, which helps to maintain blood glucose stability, it enhances satiety and promotes intestinal health (26).

**Table 1:** Chemical composition of Alisma starch, corn starch and potato starch.

	Starch content (%)	Moisture content (%)	Amylose content (%)	Amylopectin content (%)
Alisma starch	82.37 ± 0.59^c^	10.07 ± 0.35^b^	27.20 ± 0.22^a^	72.80 ± 0.22^b^
Corn starch	88.62 ± 1.51^a^	8.96 ± 0.39^b^	26.82 ± 0.27^a^	73.18 ± 0.27^b^
Potato starch	86.72 ± 0.69^b^	11.15 ± 0.10^a^	24.47 ± 0.36^b^	75.53 ± 0.36^a^

Different superscript lowercase letters in the same column mean significantly differences (*p* < 0.05).

### 3.2 Granule properties of AS

**Figure 1:**
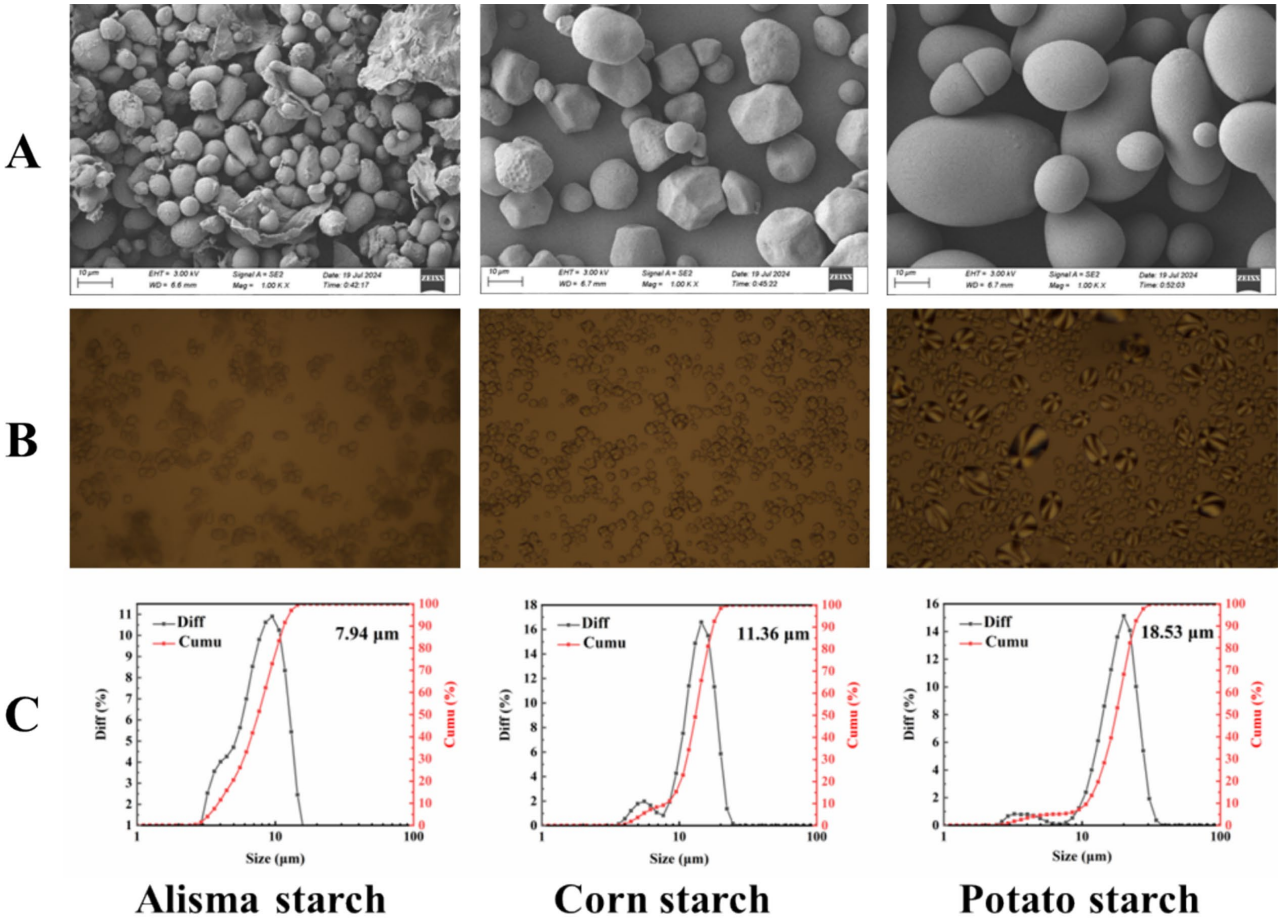
SEM images **(A)**, polarized photomicrographs **(B)**, and particle size distribution **(C)** of Alisma starch, corn starch and potato starch.

Starch generally exists in a granular state, with varying shapes and sizes of starch granules. The SEM images presented in Figure 1A indicate that AS predominantly exhibits a spherical and oval morphology, while CS mostly displays irregular shape. In contrast, PS is characterized by oval and round forms. Notably, Yu et al. (27) also reported that the appearance of AS is primarily spherical and oval. Unlike CS and PS, AS did not show polarized crosses in polarized light microscopy (Figure 1B), indicating that spherical crystal structures do not exist in AS, this absence can lead to a relatively slow rate of digestion, which has important implications for developing foods with specific health benefits (28). In addition, Figure 1C shows that the particle size of AS (7.94 μm) is significantly lower than that of CS (11.36 μm) and PS (18.53 μm). Smaller starch granules can enhance the texture of food, improve the processing efficiency of food, and improve the stability of starch (29), serving as a valuable reference for subsequent studies.

### 3.3 XRD analysis

**Figure 2:**
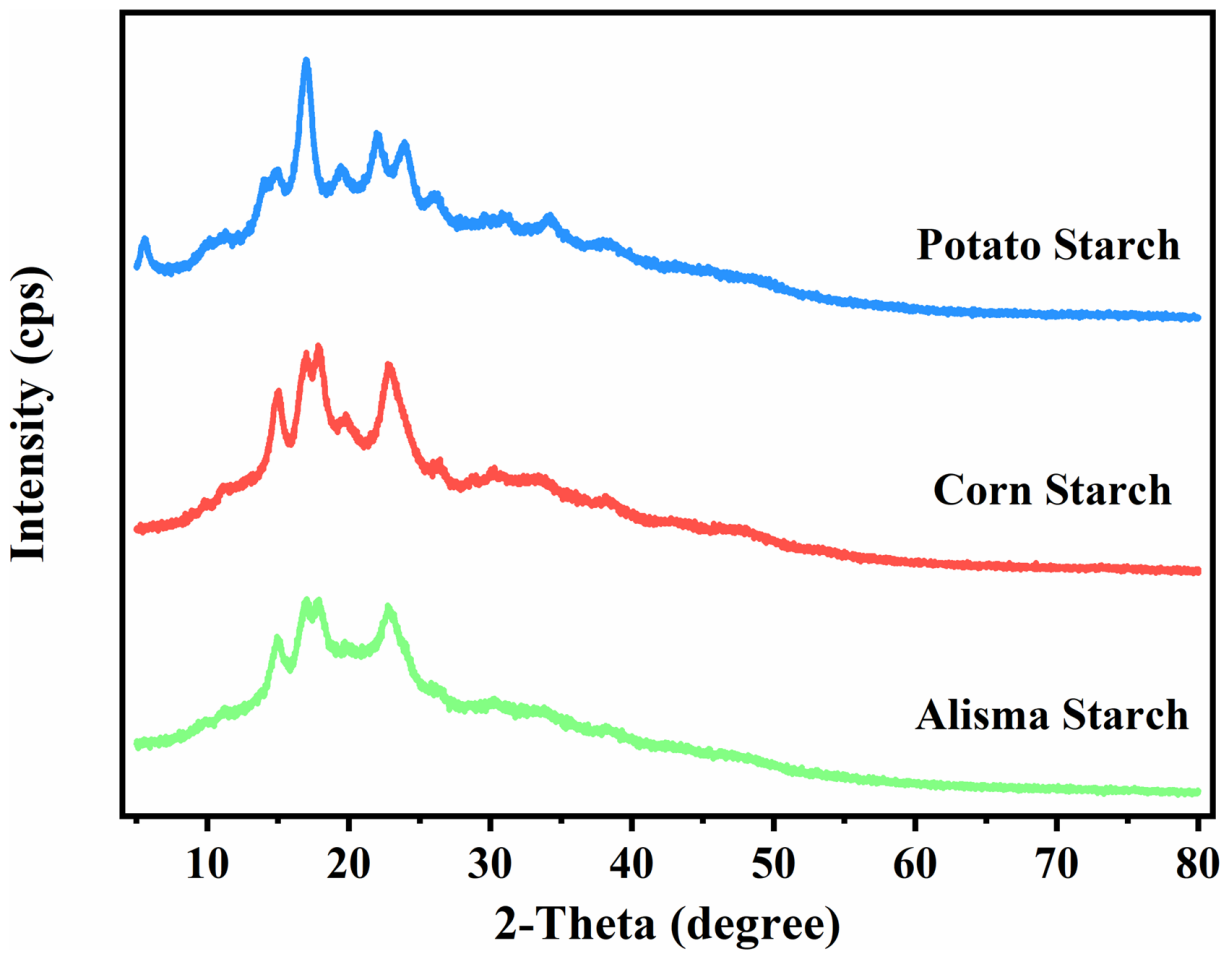
X-ray diffraction (XRD) patterns of Alisma starch, corn starch and potato starch.

Starch granules are composed of crystalline and non-crystalline regions, which alternatively constitute the granular structure of starch. The crystalline region is more compact and consists of amylopectin molecules in a double helix structure, while the non-crystalline region is easily affected by external forces and consists of amylose molecules in a loose structure (16). As shown in Figure 2, AS and CS exhibit four crystalline peaks at 15°, 17°, 18°, and 23°, indicative of the A-type starch structure, which aligns with the findings of Yu et al. (27). PS also has four crystalline peaks, with strong diffraction peaks at 15°, 17°, and 23°, respectively, and small diffraction peaks at 5.6°, characteristic of the B-type starch structure.

The crystallinity of AS, CS, and PS was determined to be 17.68, 28.10, and 22.86%, respectively, by calculating the ratio of the crystal peak area to the total diffracted area (Table 2). Differences in starch crystallinity are determined by a number of factors, including the size of the crystalline zone, the number of crystals, the chain length of the amylose, and the interactions between the double-helix structures. AS has the lowest crystallinity because amylopectin determines the ring layer size and the crystal structure, which affects the formation of crystal zones. The increase in amylose content will destroy the crystal arrangement of amylopectin and reduce the crystallinity (30). Starch with low crystallinity can reduce the formation of ice crystals during the freezing process, prevent the destruction of food tissue structure, effectively improve the freezing stability of products, and has a wide application potential in the food industry (31).

**Table 2:** X-ray diffraction (XRD) patterns analysis of Alisma starch corn starch and potato starch.

Starch name	Diffraction peak	Crystallinity	Starch type
Alisma starch	15°, 17°, 18°, 23°	17.68%	A
Corn starch	15°, 17°, 18°, 23°	28.10%	A
Potato starch	5.6°, 15°, 17°, 23°	22.86%	B

### 3.4 Pasting properties

Starch pasting represents a complex process involving a transition from an ordered to a disordered state. Initially, heating starch in excess water disrupts the crystalline regions, leading to the breaking of hydrogen bonds, followed by the diffusion of starch molecules (32). As indicated in Table 3, the pasting temperature and trough viscosity of AS (80.37 ± 0.50, 2227.67 ± 25.70) were significantly higher than those of CS (78.55 ± 0.96, 1966.33 ± 59.50) and PS (67.87 ± 0.03, 1381.33 ± 56.08) (*p* < 0.05), which indicated that AS exhibited notable resistance to heat and shear forces (33). The cold viscosity and setback viscosity of AS (3347.67 ± 36.91, 1120.00 ± 15.62) were between those of CS (2995.67 ± 36.23, 1029.33 ± 95.57) and PS (3608.33 ± 139.29, 2227.00 ± 84.88). While peak viscosity and breakdown viscosity of AS were lower, which might be due to the lower proportion of amylopectin in AS. Compared with CS and PS, AS has a higher degree of aggregation between molecular chains during the pasting process, forming a more compact microcrystalline bundle crystal structure with higher stability (32).

**Table 3:** Pasting properties of Alisma starch, corn starch and potato starch.

	Alisma starch	Corn starch	Potato starch
Pasting temperature (°C)	80.37 ± 0.50^a^	78.55 ± 0.96^b^	67.87 ± 0.03^c^
Peak viscosity (cp)	2573.33 ± 30.66^c^	2813.67 ± 3.21^b^	9695.67 ± 220.21^a^
Trough viscosity (cp)	2227.67 ± 25.70^a^	1966.33 ± 59.50^b^	1381.33 ± 56.08^c^
Cold viscosity (cp)	3347.67 ± 36.91^b^	2995.67 ± 36.23^c^	3608.33 ± 139.29^a^
Breakdown viscosity (cp)	345.67 ± 5.13^c^	847.33 ± 57.05^b^	8314.33 ± 178.16^a^
Setback viscosity (cp)	1120.00 ± 15.62^b^	1029.33 ± 95.57^c^	2227.00 ± 84.88^a^

Different superscript lowercase letters in the same row mean significantly differences (*p* < 0.05).

### 3.5 Clarity, sedimentation volume, freeze–thaw stability and syneresis of starch

**Figure 3:**
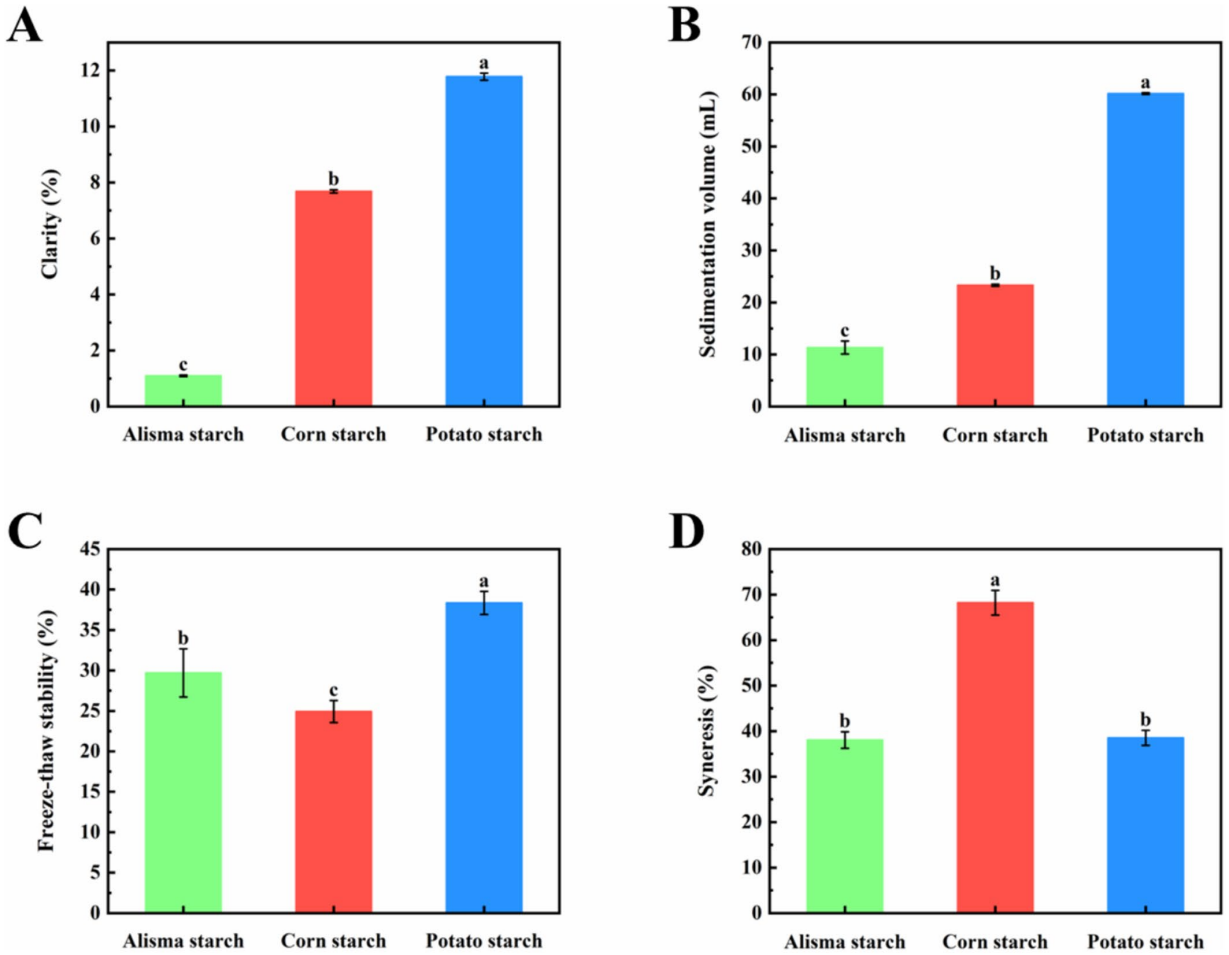
Clarity **(A)**, Sedimentation volume **(B)**, Freeze–thaw stability **(C)**, and Syneresis **(D)** of Alisma starch, corn starch, and potato starch (different letters in the same figure indicate significant differences, *p* < 0.05).

The clarity of starch paste reflects the degree of dissolution and dispersion of starch granules in water, influenced by factors such as starch granule size, amylose and amylopectin chain lengths, molecular structure, and leached amylose content. As shown in Figure 3A, the clarity of AS (1.09 ± 0.03%) is significantly lower than that of CS (7.68 ± 0.06%) and PS (11.78 ± 0.13%) (*p* < 0.05). This finding aligns with the study by Shen et al. and may be attributed to the higher amylose content in AS, which enhances molecular binding and light scattering, thereby reducing light transmittance and ultimately diminishing clarity (34).

Delamination phenomena arise upon storage of starch paste due to the unfolding of starch molecular chains, leading to the exposure of hydroxyl groups that readily recombine to form insoluble molecular microcrystalline bundles. A faster sedimentation rate indicates a higher likelihood of starch molecule recrystallization and aging reactions (35). The alteration in sedimentation volume serves as an indicator of starch coagulability strength. As depicted in Figure 3B, the sedimentation volume of AS (11.33 ± 1.26) was much smaller than that of CS (23.30 ± 0.20) and PS (60.17 ± 0.15) (*p* < 0.05), and PS had the largest sedimentation volume.

Freeze–thaw stability pertains to the capacity of starch to withstand alterations during repeated cycles of freezing and solubilization, typically manifested by variations in the syneresis rate. During the freeze–thaw process of a starch gel, molecular aggregation among starch chains induces dehydration and contraction, leading to water release from the gel structure (32). The syneresis rate serves as an indicator of the freeze–thaw property of starch, where a lower syneresis rate signifies superior freeze–thaw stability (20). Displayed in Figure 3C, the freeze–thaw stability of AS (29.69 ± 2.98%) was lower than that of PS (38.35 ± 1.42%) and higher than that of CS (24.92 ± 1.36%) (*p* < 0.05), indicating good potential for application in frozen foods.

As shown in Figure 3D, there was no significant difference in the syneresis between AS (38.03 ± 1.80%) and PS (38.50 ± 1.65%), while both were significantly lower than CS (68.23 ± 2.72%). This disparity can be attributed to the internal structure of amylose, which imparts a linear configuration during pasting, rendering it prone to precipitation from the starch paste solution with low spatial resistance and increased susceptibility to aging (34). In contrast, amylopectin, with its dendritic structure, exhibits high spatial resistance, making it less prone to precipitation from the starch paste solution and resistant to aging (35).

### 3.6 Solubility and swelling degree of starch

**Figure 4:**
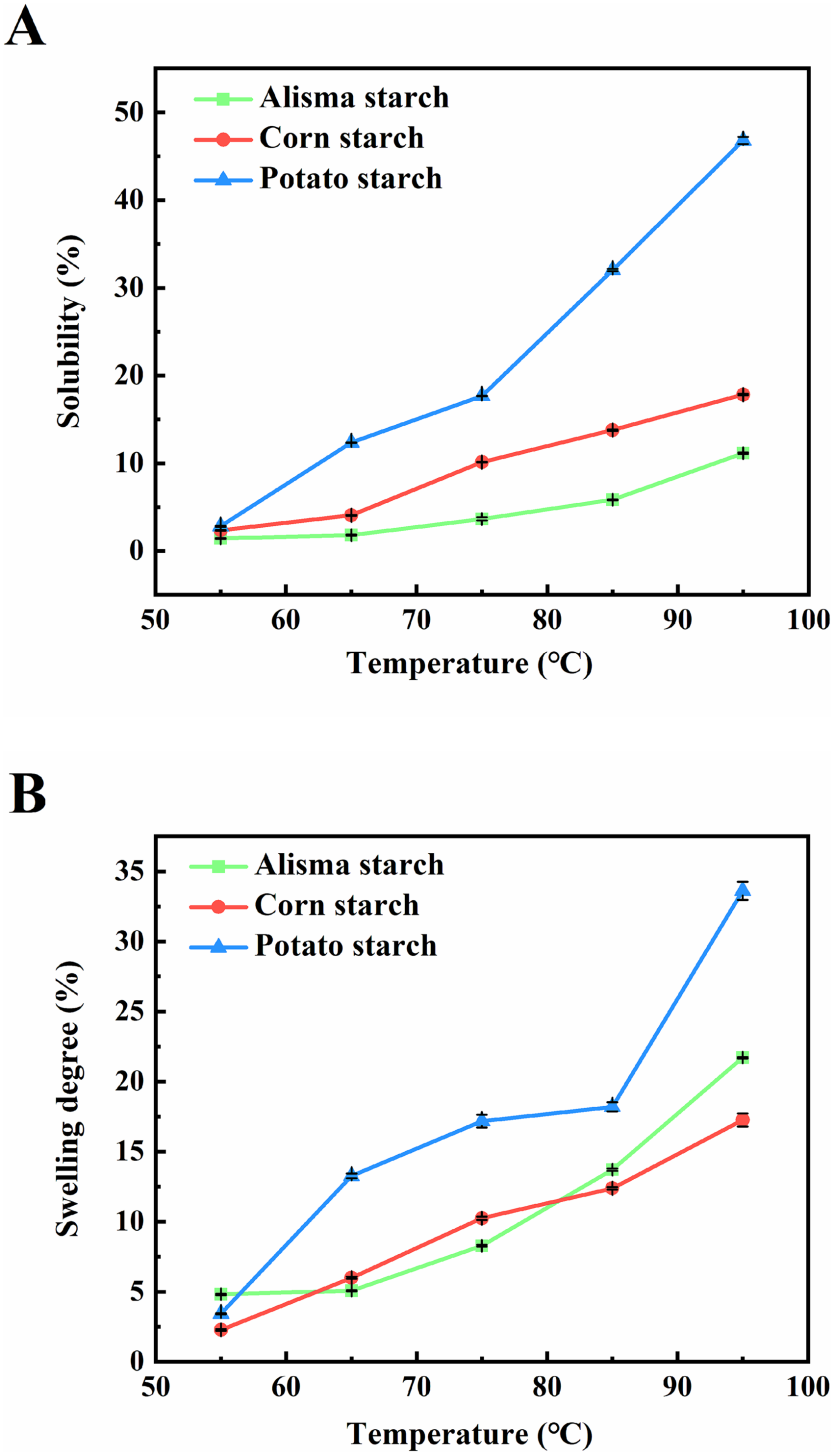
Solubility **(A)** and swelling degree **(B)** of Alisma starch, corn starch, and potato starch.

The solubility and swelling degree of starch reflect the characteristics of starch granules such as size, morphology and molecular weight, as well as the interaction between water and starch. Solubility refers to the degree of dissolution of starch during swelling, and swelling degree refers to the water holding capacity of starch. Illustrated in Figures 4A,B, the solubility and swelling degree of the three starches increased with increasing temperature. Solubility and swelling degree increased rapidly at 85–95°C, a phenomenon consistent with findings reported in previous studies on starches derived from diverse sources (36). The elevation in temperature disrupts the crystalline regions of starch, facilitating the unfolding of starch molecular chains and exposing additional hydroxyl groups capable of forming hydrogen bonds with water molecules. This process enhances the solubility of starch and promotes water absorption by starch granules, leading to an escalation in granule size and swelling degree. Upon surpassing the pasting temperature of starch, rapid swelling and disintegration of starch granules occur, causing the dissolution of starch molecules and a sharp surge in solubility. Between 65 and 95°C, the solubility of AS is significantly lower than that of CS and PS, demonstrating a typical two-stage swelling process for all three starches. This observation is consistent with findings reported by Lv et al. (35). Notably, AS displayed a higher swelling rate than CS but lower than PS within the 85–95°C range. Throughout the process, PS consistently demonstrated superior solubility and swelling degree in comparison to AS and CS. This distinction may be attributed to the larger granule size of PS and the presence of phosphate groups on amylopectin molecules, factors known to enhance solubility and swelling degree. Moreover, Singh and Singh (37) found that the possible lack of amylose-lipid complexes in PS likely reduced intermolecular interactions within starch molecules, facilitating easier swelling and dissolution upon hydration.

### 3.7 Gel strength of starch

**Figure 5:**
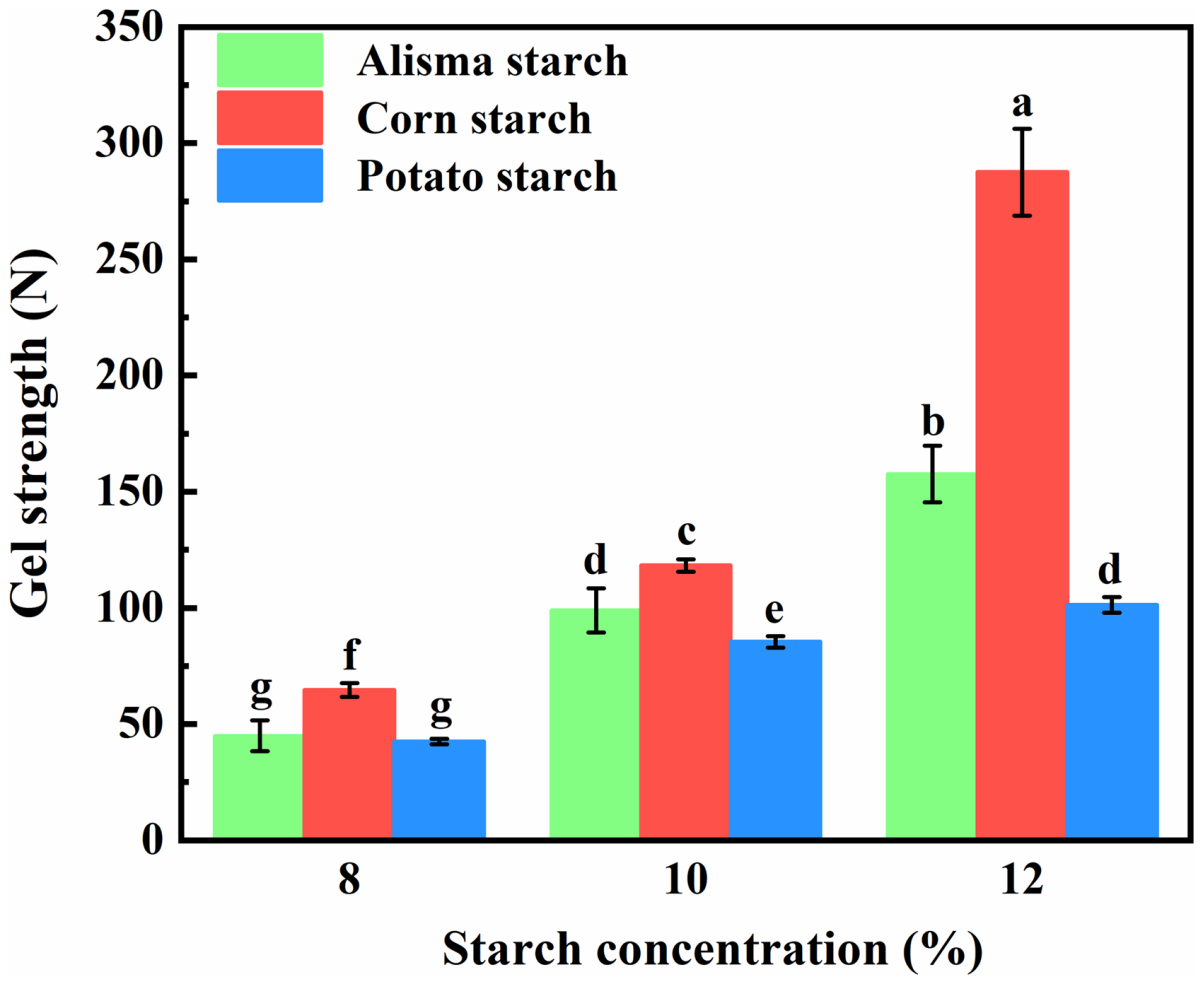
Gel strength of Alisma starch, corn starch and potato starch (different letters in the same figure indicate significant differences, *p* < 0.05).

The cooling process following the heating pasteurization of starch for starch gel formation involves the molecular reorganization of starch. Upon heating, the thermal movement loosens the starch molecular chains; however, upon cooling, amylose and amylopectin molecules reorganize and aggregate via intermolecular hydrogen bonding to establish a gel structure (28). Illustrated in Figure 5 are the gel strengths of AS, CS, and PS at varying concentrations. The gel strength of all three starches exhibited a gradual increase with rising starch concentration. Among the starches, CS demonstrated the most pronounced enhancement in gel strength, followed by AS and PS, respectively. At a starch concentration of 8%, the gel strengths of AS (44.98 ± 6.62) and PS (42.48 ± 1.14) did not exhibit significant disparities and were lower than that of CS (64.69 ± 3.03). Upon increasing the starch concentration to 10 and 12%, the gel strengths of AS fell between those of CS and PS. Notably, there was no notable difference in gel strength between AS (98.95 ± 9.55) at a 10% concentration and PS (101.33 ± 3.33) at a 12% concentration. This observation may be attributed to the amylose content, as the linear structure of amylose molecules facilitates their reorganization and aggregation, leading to the formation of increased intermolecular hydrogen bonds that serve as cross-linking points. These bonds contribute to the establishment of a robust network structure, thereby enhancing the gel strength (38).

## 4 Conclusion

The starch content of AS (82.37 ± 0.59%) was lower than that of CS and PS, while exhibiting a higher amylose content (27.20 ± 0.22%), smaller particle size (7.94 μm), spherical and elliptical shape, which belonged to the A-type starch. Compared with CS and PS, AS had higher heat resistance as well as lower clarity and sedimentation volume. The syneresis of AS was not significantly different from that of PS, and its freeze–thaw stability was between CS and PS. Furthermore, the gel strength of all three starches increased gradually with increasing starch concentration. There was no significant difference in gel strength between AS at a 10% concentration and PS at a 12% concentration. Based on the research findings, future studies should delve into the heat resistance mechanisms of AS to explore its potential applications in high-temperature processed foods. Additionally, we should focus on the unique physicochemical properties of AS to broaden its range of applications in food thickening, stability, health, and other aspects. This will facilitate the comprehensive utilization of AS resources.

## Data Availability

The original contributions presented in the study are included in the article/supplementary material, further inquiries can be directed to the corresponding author.
